# The genetic causal effect of hand grip strength on osteoporosis and falling risk: a Mendelian randomization study

**DOI:** 10.3389/fendo.2024.1433805

**Published:** 2024-10-02

**Authors:** Yanhua Ma, Jingtao Qiao, Zhenxing Wang, Qi Pan, Lixin Guo

**Affiliations:** ^1^ Department of Endocrinology, Beijing Hospital, National Center of Gerontology, Institute of Geriatric Medicine, Chinese Academy of Medical Sciences, Beijing, China; ^2^ Graduate School of Peking Union Medical College, Chinese Academy of Medical Sciences, Beijing, China; ^3^ Peking University Fifth School of Clinical Medicine, Beijing, China

**Keywords:** hand grip strength, osteoporosis, falling risk, single nucleotide polymorphisms, Mendelian randomization

## Abstract

**Background:**

Patients with osteoporosis (OP) are often associated with decreased hand grip strength and increased risk of falling. It remains unclear whether there is a genetic causal between hand grip strength and OP, falling risk.

**Methods:**

The Mendelian randomization study was used to investigate the genetic causal effect of low hand grip strength on total body bone mineral density (BMD) at different ages, OP, and falling risk. Genes for low hand grip strength, total body BMD at different ages, OP, and falling risk were obtained from published genome-wide association studies. Inverse variance weighted, MR‐Egger, and weighted median were applied to perform the MR analysis. The Cochran’s Q test, MR‐Egger intercept test, MR-PRESSO global test, and leave-one-out analysis were used to detect the pleiotropy or heterogeneity.

**Results:**

The results showed strong evidence that low hand grip strength was positively associated with OP (OR: 1.006, 95% CI: 1.003-1.010; P= 0.0001) and falling risk (OR: 1.069, 95% CI: 1.013-1.129; P= 0.0160), and could not directly affect the different ages of total body BMD (P> 0.05). There was no heterogeneity or horizontal pleiotropy in the sensitivity analysis (all P> 0.05).

**Conclusion:**

The study found a positive causal relationship between low hand grip strength and higher risk of OP and falling, which should be taken into account in the development of future prevention and screening strategies for OP and falling.

## Graphical Abstract

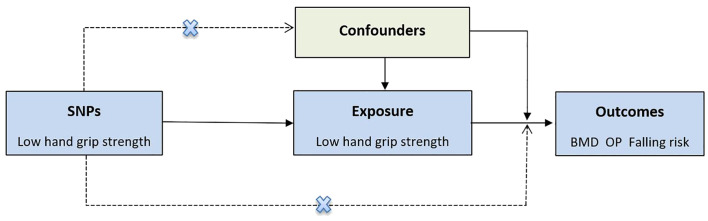

## 1 Introduction

As the world’s population ages, osteoporosis (OP) is becoming one of the most prevalent metabolic bone diseases, increasing the risk of insufficiency fractures (1, 2). Over the last 12 years, the prevalence of OP in China has increased, affecting more than one-third of the population over the age of 50 (3). Osteoporotic fracture is the most serious consequence of OP, which has a significant financial impact on society (4). The burden of the disorder may therefore be lessened by identifying risk factors for OP, which will enable us to identify those who are at risk and create intervention techniques for prevention or early treatment (5).

Sarcopenia is a syndrome characterized by progressive and pervasive loss of skeletal muscle mass and strength (6). According to research data, the current prevalence of sarcopenia in the community is at 1-33% (7), sarcopenia affects around 50 million individuals globally, and that number is expected to rise to 200 million in the next 40 years (6). Sarcopenia can lead to mobility disorders (8), compromise life quality (9), and increase personal, social, and economic burdens (10).

Sarcopenia and OP are two disorders with similar risk factors and biological pathways (11). Bone and muscle interact closely with one another physically, chemically, and metabolically (12). OP and sarcopenia frequently coexist (13–15), and are strongly associated with frailty, falls, fractures, hospitalizations, and mortality (16–18). Current evidence also suggests that sarcopenia may be an independent predictor of low BMD and OP (13). Decreased hand grip strength is an important part of the diagnostic criteria for sarcopenia (19). Hand grip strength is the most preferable method of measuring muscle strength because it is a simple, noninvasive indicator of muscle strength and is ideal for clinical use (20). In recent years, some studies have shown that low hand grip strength could predict decreased bone mineral density (BMD) (13, 21–23), the increased prevalence of OP (24, 25), and falling risk (26, 27), but the findings are inconsistent, and the limitations of observational studies make it unclear whether these associations are confounding or causal (28). Further research at the genetic level is required in order to fully understand the significance of these associations for disease prevention and screening.

Mendelian randomization (MR) is a method based on genome-wide association study (GWAS) data, where genetic variation is used as an instrumental variable (IV) to infer the specific effect of exposure on outcome (29). Genes are randomly assigned to the offspring without being subject to confounding factors because gamete formation follows Mendelian laws (30). To the best of our knowledge, no similar MR studies have been conducted to explore the causal relationship between hand grip strength and OP and fall risk. The MR study aimed to investigate the causal effect of low hand grip strength on the total body BMD at different ages, the prevalence of OP, and falling risk.

## 2 Materials and methods

### 2.1 Data sources

Single nucleotide polymorphisms (SNPs) were used as IVs in the MR investigation to demonstrate a causal relationship between low hand grip strength and total body BMD, OP, falling risk. The summary statistics of SNPs related to low hand grip strength, total body BMD at different ages, OP, and falling risk were extracted from the GWAS database (https://gwas.mrcieu.ac.uk), which is publicly available, and the detailed information is shown in **Supplementary Table S1**. Since this study was based on published data, no ethical approval or informed consent was required.

### 2.2 Selection of genetic instruments

In the MR analysis, IVs must meet the three key assumptions (30, 31). First, SNPs are strongly associated with low hand grip strength. Second, SNPs shouldn’t be associated with any confounders. Third, SNPs affect the outcome only via low hand grip strength (Graphical Abstract).

With P < 5×10^-8^ serving as the screening condition, SNPs for low hand grip strength were selected as IVs based on published data. At the same time, we excluded SNPs that were in linkage disequilibrium status (R^2^ < 0.001, aggregation window = 10,000kb) to ensure independence. Finally, we calculated the R^2^ and F statistic to evaluate the bias of the weak IVs using the following formula: R^2^ = 2 × EAF × (1-EAF) × β^2^, F = R^2^ (N-K-1)/[(1-R^2^)], where N is the sample size, K is the number of IVs, and SNPs with F greater than 10 were further analyzed (32). Following a comprehensive screening process, the residual SNPs were employed in further investigations.

### 2.3 MR analysis

The random-effects inverse variance weighted (IVW) was the primary statistical method, which was used to analyze the primary causal inference of the effect of low hand grip strength on BMD, OP, and falling risk. To improve the confidence of the results, we used two additional MR methods, the weighted median and MR-Egger methods for causal association assessments. In addition, we performed a series of sensitivity analyses to assess the reliability of the MR results. The Cochran’s Q test was used to detect heterogeneity of IVs. The MR-Egger intercept test and MR-PRESSO global test were used to examine the horizontal pleiotropy, and a leave-one-out sensitivity analysis was performed to assess the stability of the MR results.

### 2.4 Statistical analysis

The “TwoSampleMR” and “MRPRESSO” packages of the R software (version 4.3.1) were performed to implement all statistical analyses. The MR results were represented by odds ratios (ORs) and 95% confidence intervals (CIs). P < 0.05 was statistically significant.

## 3 Results

### 3.1 Genetic variables for low hand grip strength

As shown in **Supplementary Table S2**, in our MR study, seventeen SNPs were chosen as IVs for low hand grip strength from published data, and the F of all SNPs was greater than 10, no bias was found for weak IVs.

### 3.2 The influence of genetically predicted low hand grip strength on total body BMD at different ages

**Figure 1:**
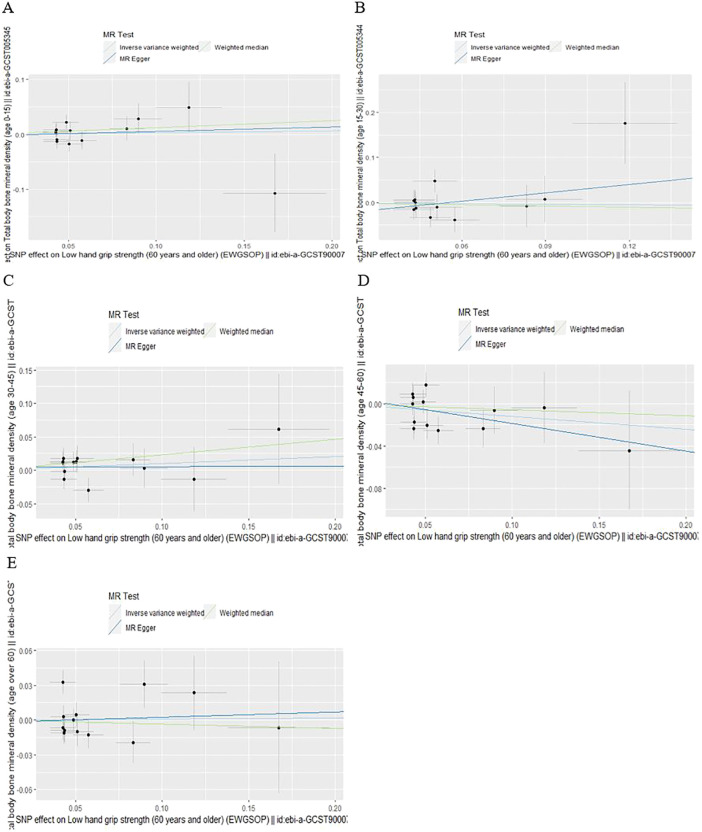
Scatter plot of the causal effect of low hand grip strength on different ages of total body bone mineral density **(A)** Age 0-15, **(B)** Age 15-30, **(C)** Age 30-45, **(D)** Age 45-60, **(E)** Age over 60.

**Figure 2:**
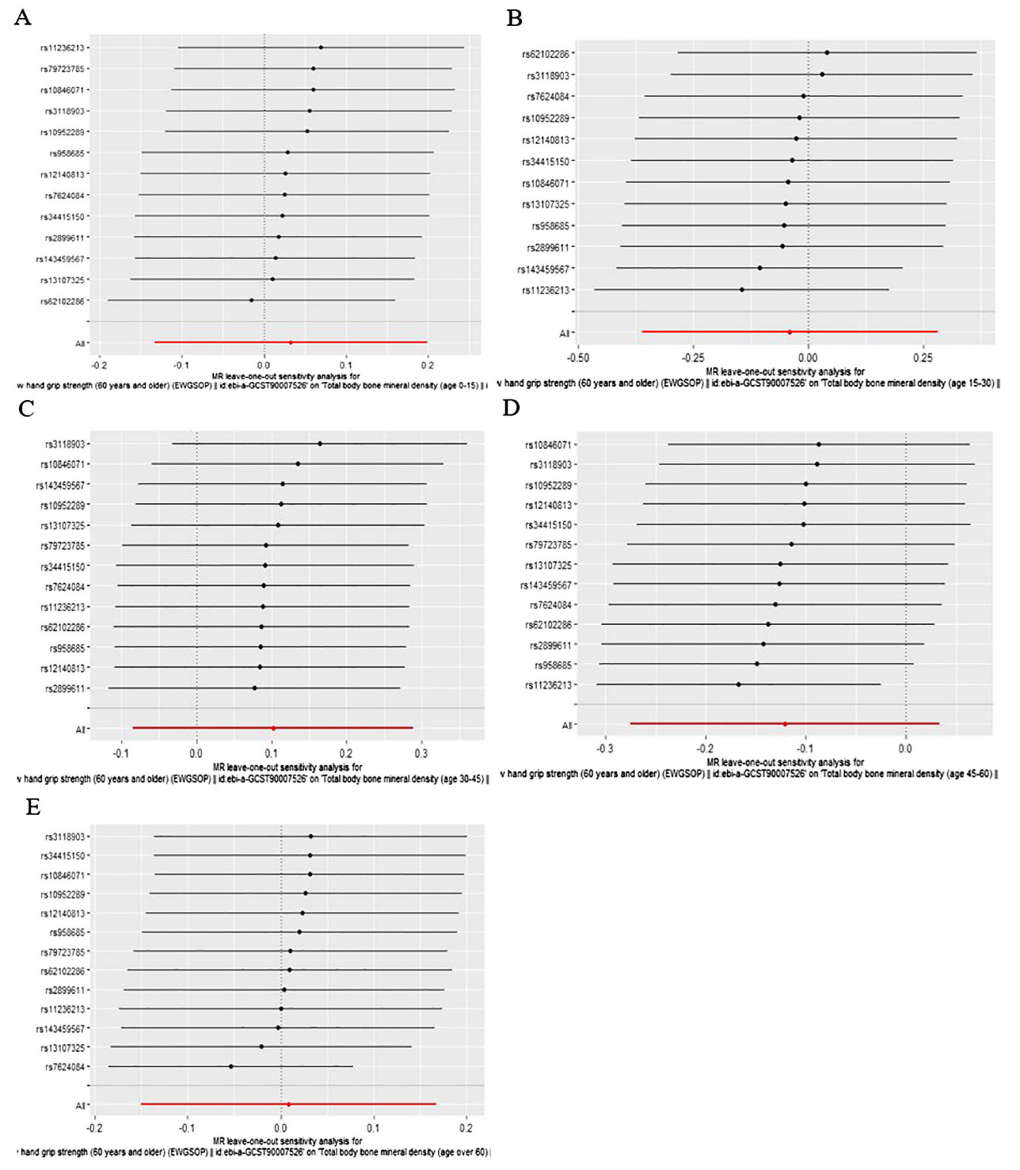
Leave-one-out plot of the causal effect of low hand grip strength on different ages of total body bone mineral density **(A)** Age 0-15, **(B)** Age 15-30, **(C)** Age 30-45, **(D)** Age 45-60, **(E)** Age over 60.

According to IVW analysis, the MR results indicated low hand grip strength could not directly affect the different ages of total body BMD (BMD age 0-15: OR = 1.03, 95% CI: 0.88-1.22, p = 0.698; BMD age 15-30: OR = 0.96, 95% CI: 0.70-1.33, p = 0.808; BMD age 30-45: OR = 1.11, 95% CI: 0.92-1.33, p = 0.284; BMD age 45-60: OR = 0.89, 95% CI: 0.76-1.03, p = 0.125; BMD age over 60: OR = 1.01, 95% CI: 0.86-1.18, p = 0.916), and the results were also confirmed by the MR-Egger regression and weighted median methods (all P > 0.05), which are presented in **Table 1**, **Figure 1** and **Figure 2**.

**Table 1:** MR estimates of the causal association between low hand grip strength, total body bone mineral density at different ages, the risk of osteoporosis, and falling.

Outcome	Methods	OR	95%CI	P-value
Total body bone mineral density (age 0-15)	IVW	1.03	0.88-1.22	0.698
	WM	1.13	0.89-1.44	0.298
	MR Egger	1.09	0.59-2.00	0.798
Total body bone mineral density (age 15-30)	IVW	0.96	0.70-1.33	0.808
	WM	0.92	0.60-1.41	0.695
	MR Egger	1.85	0.45-7.56	0.412
Total body bone mineral density (age 30-45)	IVW	1.11	0.92-1.33	0.284
	WM	1.26	0.98-1.61	0.069
	MR Egger	1.00	0.52-1.93	0.994
Total body bone mineral density (age 45-60)	IVW	0.89	0.76-1.03	0.125
	WM	0.95	0.78-1.15	0.575
	MR Egger	0.77	0.44-1.34	0.372
Total body bone mineral density (age over 60)	IVW	1.01	0.86-1.18	0.916
	WM	0.96	0.80-1.16	0.691
	MR Egger	1.05	0.58-1.89	0.883
Osteoporosis	IVW	1.01	1.00-1.01	<0.001
	WM	1.01	1.00-1.01	0.001
	MR Egger	1.00	0.99-1.02	0.710
Falling risk	IVW	1.07	1.01-1.13	0.016
	WM	1.05	0.98-1.12	0.200
	MR Egger	1.14	0.95-1.37	0.174

MR, Mendelian randomization; OR, odds ratio; 95%CI, 95% confidence interval; IVW, inverse variance weighted; WM, weighted median.

### 3.3 The influence of genetically predicted low hand grip strength on OP

**Figure 3:**
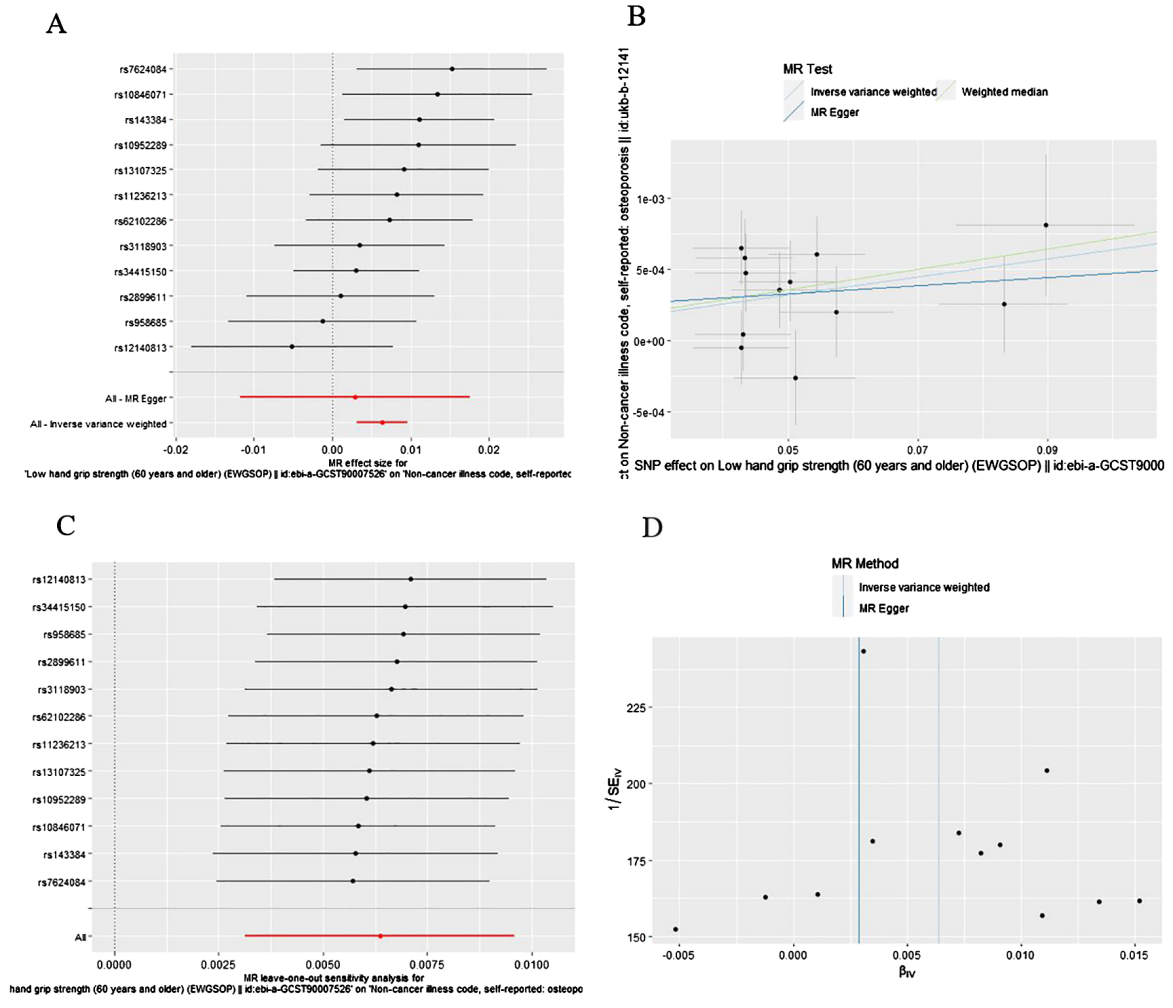
The causal effect of low hand grip strength on osteoporosis risk. **(A)** Forrest plot, **(B)** Scatter plot, **(C)** Leave-one-out plot, **(D)** Funnel plot.

As presented in **Table 1**, **Figure 3**, the MR results of IVW analysis indicated low hand grip strength could directly affect the OP (OR: 1.006, 95% CI: 1.003-1.010, P= 0.0001), and the results were also confirmed by the weighted median methods (OR = 1.007, 95% CI: 1.003-1.012, P= 0.421).

### 3.4 The influence of genetically predicted low hand grip strength on falling risk

**Figure 4:**
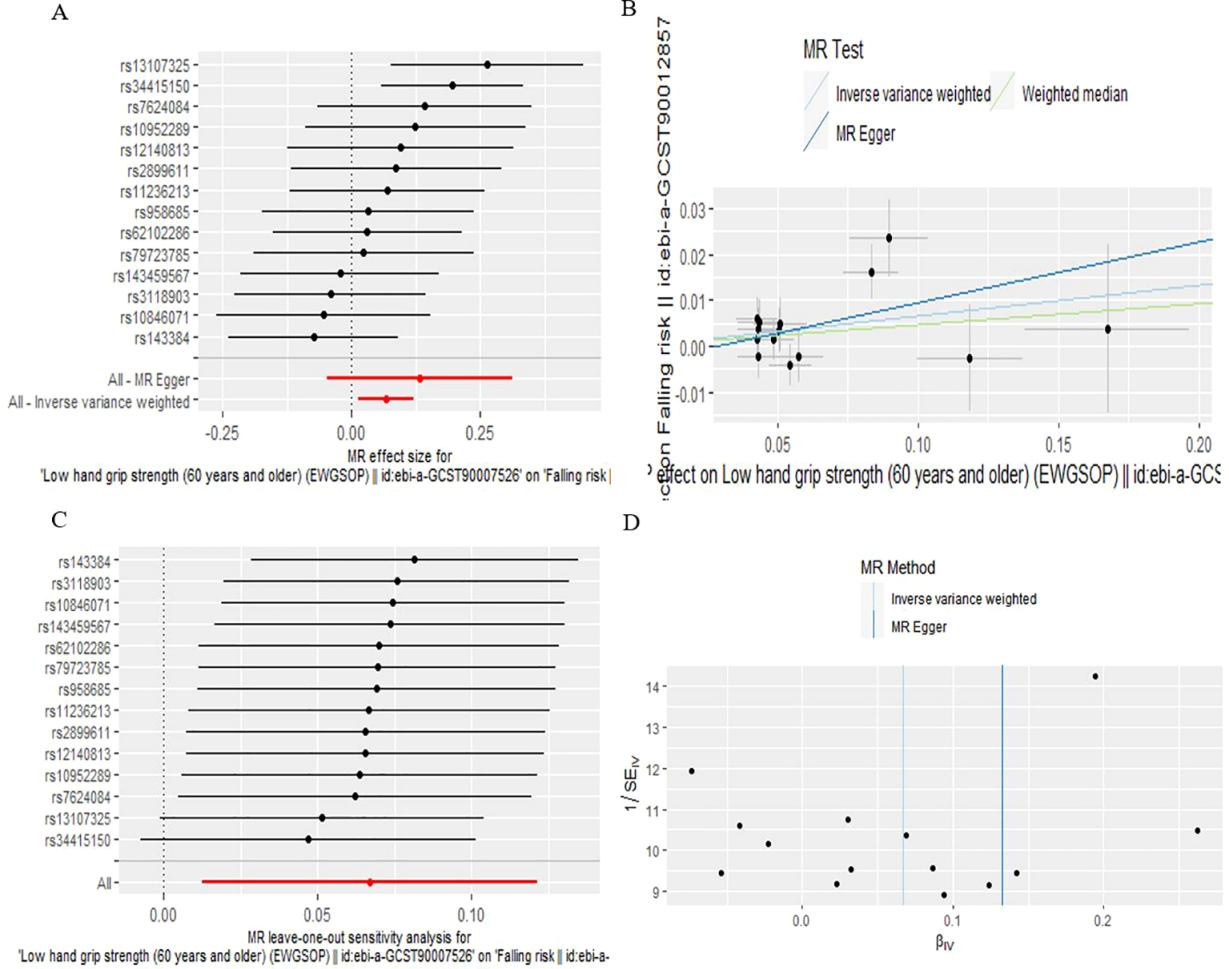
The causal effect of low hand grip strength on falling risk. **(A)** Forrest plot, **(B)** Scatter plot, **(C)** Leave-one-out plot, **(D)** Funnel plot.

According to IVW analysis, the MR results indicated low hand grip strength could directly affect the falling risk (OR: 1.069, 95% CI: 1.013-1.129; P=0.0160), which are presented in **Table 1**, **Figure 4**.

### 3.5 Results of the sensitivity analysis

As is shown in **Table 2**, all p values for Cochran’s Q test analysis were more than 0.05, indicating no heterogeneity in the study. Also, the P values of the MR‐Egger intercept test and MR-PRESSO global test were all greater than 0.05, indicating no horizontal pleiotropy (**Table 2**). Additionally, each SNP was gradually removed by using the leave-one-out method, and the results were all the same as the original results, which showed the results of the study to be of heightened reliability (**Figures 2**–**4**).

**Table 2:** Sensitivity analysis of the causal association between low hand grip strength, total body bone mineral density at different ages, the risk of osteoporosis, and falling.

Exposure	Outcome	Cochran Q test		MR‐Egger		MR-PRESSO
		Q-value	P-value	Intercept	P-value	P-value
Low hand grip strength	Total body bone mineral density (age 0-15)	11.63	0.475	-0.0027	0.873	0.497
	Total body bone mineral density (age 15-30)	12.25	0.345	-0.0338	0.371	0.356
	Total body bone mineral density (age 30-45)	8.22	0.768	0.0055	0.762	0.760
	Total body bone mineral density (age 45-60)	15.59	0.211	0.0077	0.611	0.215
	Total body bone mineral density (age > 60)	18.81	0.093	-0.0020	0.901	0.101
	Osteoporosis	11.48	0.404	0.0002	0.642	0.458
	Falling risk	15.03	0.305	-0.0038	0.466	0.298

## 4 Discussion

Exploring the causal relationship between hand grip strength and OP and falling risk is important for the prevention and screening of OP and falling. Previous studies are contradictory and have limitations in study design. Our study was conducted by a MR analysis method utilizing publicly available large-scale GWAS summary data, which ultimately found that genetic susceptibility to hand grip strength directly altered the risk of OP and falling. To our knowledge, this is the first MR study exploring the causal effect of hand grip strength on OP and falling risk.

In 2019, the revision of the European consensus on the definition and diagnosis of sarcopenia suggested that hand grip strength could be an initial assessment tool for sarcopenia, which could effectively help to identify cases in clinical practice (19). This study used hand grip strength as a representative indicator of sarcopenia to provide a more specific analysis of the effect of sarcopenia on BMD, OP, and fall risk at the genetic level.

Some previous studies have examined the association between hand grip strength on BMD. A retrospective analysis of 1,850 participants aged 40-80 years found that grip strength was associated with increased femoral neck and total lumbar spine BMDs in men (P < 0.001, P = 0.005), after adjusting for age, ethnicity, body mass index, use of female hormones, smoking habit, drinking habit, family history of OP, use of calcium and vitamin D supplements, physical activity, serum calcium, and phosphorus levels, with the same results obtained in both premenopausal (P = 0.040, P = 0.014) and postmenopausal women (P = 0.016, P = 0.012) (24). Besides, a study analyzed the relationship between hand grip strength and BMD in 1,427 adolescent students in Chile (750 males and 677 females, aged between 11.0 and 18.9 years) and found that grip strength was positively correlated to BMD in adolescents (25). Interestingly, some studies in recent years have found inconsistent results. A cross-sectional study analyzing the relationship between grip strength and BMD in 318 men (age range 33-92 years) and 203 women (age range 41-90 years) in China, showed that hand grip strength was not associated with BMD in men (28). In addition, a study included 234 male participants and found that there was no predictive value of hand grip strength for BMD of the lumbar spine or femoral neck (33). Besides, the study of Robert et al. (34) analysis of the Framingham Offspring study, which included 508 men and 651 women (aged 50 years and older), found that greater hand grip strength was associated with larger bone size and greater bone strength at the distal radius, which suggested that loading by muscles may not affect BMD or microarchitecture, thus the positive relation between muscle strength and bone strength may be driven primarily by bone size. Consistent with recent findings, our study found no causal relationship between low hand grip strength and total body BMD at different ages, at least at the genetic level. Although the previous MR studies found a site-specific effect of handgrip strength on lumbar spine BMD, stratified analyses by age have not been further explored (35, 36). The inconsistencies between the results of previous studies may be due to differences in the characteristics of the study population, such as race, age, gender, BMI, co-morbidities, smoking alcohol drinking status. In addition, the varying degree of standardization in the methodology and operation of the tests of grip strength and BMD in different observational studies.

In addition, recent observational studies have examined the effect of hand grip strength on OP risk. A cross-sectional descriptive study analyzing 1,168 Chinese individuals aged ≥ 60 years (mean age: 66.9 ± 6.2 years; men, n = 516; women, n = 652) found that higher grip strength was associated with a lower risk of OP (P = 0.023) (37). Besides, a retrospective study analyzed body composition data from 17,891 African American, Caucasian, and Chinese subjects and found that sarcopenia was associated with low whole-body BMDs and OP (13). Muscle weakness is one of the major predictors of falls. What’s more, recent studies have also shown that hand grip strength, a marker of sarcopenia, is associated with fall risk (27). A retrospective study that included 3,334 Swedish 70-year-olds found that patients diagnosed with sarcopenia exhibited worse BMD and were at higher risk for falls than those with suspected or no sarcopenia (P < 0.05) (38). Francesco et al.’s study (39) evaluated the relationship between sarcopenia and 2-year risk of falls in individuals aged 80 years or older, and after adjusting for confounding factors, it was found that participants with sarcopenia had a higher risk of incident falls compared with non-sarcopenic subjects (adjusted hazard ratio [HR], 3.23; 95% CI, 1.25-8.29). A cross-sectional study analyzing data from 349 patients with OP (median age 77.0 years) found that low hand grip strength is independently and positively associated with fall risk in older women with OP (26). In our study, we found a positive causal relationship between low hand grip strength and prevalence of OP and falling risk, at least at the genetic level, which was in line with the above findings. The management of sarcopenia should be regarded as a key point in the prevention and treatment of OP (40). In addition, sarcopenia also plays an important role in preventing falls in the elderly (39). It’s worth noting that in our study, low hand grip strength had no effect on total body BMD at different ages but increased the risk of OP. In addition to BMD, bone strength can be affected by bone microarchitecture, also known as bone mass, the latter of which can currently be assessed by testing trabecular bone score (TBS) for bone microarchitecture, and a recent study has also found that the low hand grip strength was positively associated with low TBS (28).

Bone and skeletal muscle are integral organs and the coupling between them is considered to be primarily mechanical (41). In addition to the direct effects of weight-bearing, physical activity is the main physiological stimulus that promotes skeletal anabolism and/or catabolism through actin production and secretion (41). However, skeletal muscle can also influence skeletal homeostasis in a non-mechanical way, i.e., through its endocrine activity, actin secreted by skeletal muscle has not only an autocrine function in regulating muscle metabolism but also a paracrine or endocrine regulatory function in distant organs and tissues such as bone and adipose tissue (41, 42). Age-related skeletal muscle decline may lead to bone loss through biomechanical stimulation and decreased growth factors, ultimately leading to the development of OP (43). As a result, patients with sarcopenia have an increased likelihood of developing OP, and some experts have suggested that the two disorders should be combined into a single disease called “osteosarcopenia” (12).

The strengths of the study include the analysis of a large sample size of GWAS data and the stratification of different ages of total body BMD. Additionally, compared to observational studies, MR methods can strengthen the evidence for causal inferences due to the strength of the MR study (44). Nevertheless, our study has some limitations. Firstly, the OP cases were from self-reported OP patients in the UK Biobank. Since disease reporting accuracy varies, there is a risk that OP cases will be misdiagnosed or underdiagnosed. Secondly, we must recognize that there are some limitations to the key assumptions of MR, as it is difficult to ensure that the exposure-outcome relationship is free of any confounders or potential pleiotropic effects. In addition, the GWAS data of the total body BMD lacked gender stratification, preventing a more detailed analysis of the causal relationship between hand grip strength and BMD in different gender subgroups. Besides, we were unable to perform stratified MR analyses based on subtypes of OP, which would have helped to improve the accuracy of the study. It is worth noting that hand grip strength is closely related to quality of life, especially among the elderly and hospitalized population (45). However, in our study, we were unable to assess the quality of life of the included participants, which is a confounding factor that needs to be considered. What’s more, the elderly population may suffer from malnutrition and malabsorption, which are related to OP and muscle strength (46), and can also affect the grip strength test results of participants, which need to be carefully considered. Furthermore, although the MR method was used in this study to assess the causal relationship, it presupposes that there is a linear relationship between exposure and outcome, otherwise, this method is not applicable, so prospective cohort data are still needed to validate this in the future.

In summary, our study provides genetic evidence to support a causal association between low hand grip strength and OP, fall risk. Hand grip strength measurement is a simple, cost-effective, and easy-to-administer assessment method for identifying people at high risk for OP and falls, which should be taken into account in the development of future prevention and screening strategies for the disease.

## Data Availability

The original contributions presented in the study are included in the article/**Supplementary Material**. Further inquiries can be directed to the corresponding authors.
