# Supplementary material for: The genetic causal effect of hand grip strength on osteoporosis and falling risk: a Mendelian randomization study
Source: Front Endocrinol (Lausanne). 2024 Oct 2;15:1433805. doi: 10.3389/fendo.2024.1433805 (PMC11479888; doi:10.3389/fendo.2024.1433805)
Supplement: Supplementary file 1 [file Table1.docx]

Supplementary Material

# Supplementary Tables

**Table S1 Overview of GWAS datasets for low hand grip strength, total body bone mineral density** **at different ages, the risk of osteoporosis and falling**

| **Phenotype** | **Population** | **Cohort** | **Sample size** | **nSNPs** | **Value type** |
| --- | --- | --- | --- | --- | --- |
| Low hand grip strength | European | UK Biobank | 256,523 | 9,336,415 | Binary |
| Total body bone mineral density (age 0-15) | Mixed | GWAS meta-analysis | 11,807 | 9,351,693 | Continuous |
| Total body bone mineral density (age 15-30) | Mixed | GWAS meta-analysis | 4,180 | 8,509,502 | Continuous |
| Total body bone mineral density (age 30-45) | Mixed | GWAS meta-analysis | 10,062 | 9,656,698 | Continuous |
| Total body bone mineral density (age 45-60) | Mixed | GWAS meta-analysis | 18,805 | 10,304,110 | Continuous |
| Total body bone mineral density (age over 60) | Mixed | GWAS meta-analysis | 22,504 | 11,932,096 | Continuous |
| Osteoporosis | European | UK Biobank | 462,933 | 9,851,867 | Binary |
| Falling risk | European | UK Biobank | 451,179 | 7,720,247 | Binary |

Abbreviations: GWAS: genome-wide association study, BMD: bone mineral density, OP: osteoporosis, nSNPs: Number of SNPs.

**Table S2 Instrument variables of low hand grip strength**

| **SNP** | effect allele | another allele | BETA | EAF | SE | P value | F statistics |
| --- | --- | --- | --- | --- | --- | --- | --- |
| rs12140813 | T | C | 0.0511 | 0.1856 | 0.0094 | 4.76E-08 | 203 |
| rs958685 | A | C | -0.0428 | 0.5142 | 0.0074 | 6.52E-09 | 235 |
| rs7624084 | C | T | -0.0428 | 0.4423 | 0.0074 | 8.51E-09 | 232 |
| rs13107325 | T | C | 0.0897 | 0.0739 | 0.0138 | 7.42E-11 | 283 |
| rs34415150 | G | A | 0.0833 | 0.178 | 0.0099 | 4.42E-17 | 522 |
| rs185320691 | C | G | 0.0913 | 0.1047 | 0.0146 | 3.84E-10 | 402 |
| rs10952289 | C | T | -0.0435 | 0.3436 | 0.0078 | 2.10E-08 | 219 |
| rs11236213 | A | G | -0.0504 | 0.3136 | 0.008 | 3.01E-10 | 281 |
| rs10846071 | T | C | 0.0433 | 0.3917 | 0.0075 | 7.32E-09 | 229 |
| rs34464763 | A | T | 0.0544 | 0.3943 | 0.0086 | 3.15E-10 | 363 |
| rs3118903 | A | G | 0.0575 | 0.2185 | 0.0088 | 6.71E-11 | 290 |
| rs2899611 | G | T | 0.0431 | 0.5049 | 0.0074 | 6.01E-09 | 238 |
| rs8061064 | A | T | 0.0407 | 0.4627 | 0.0074 | 3.55E-08 | 211 |
| rs143459567 | T | C | 0.1185 | 0.0386 | 0.0189 | 3.41E-10 | 268 |
| rs62102286 | G | T | -0.0487 | 0.4359 | 0.0074 | 5.49E-11 | 300 |
| rs79723785 | C | T | 0.1674 | 0.0165 | 0.0293 | 1.16E-08 | 234 |
| rs143384 | G | A | -0.0545 | 0.4088 | 0.0075 | 4.47E-13 | 369 |

Abbreviations: SNP is the rsID of genetic variants; EAF is the effect allele frequency; BETA is the effect size of effect the allele on the exposure; SE is the standard error of beta.
