# Supplementary material for: The genetic causal effect of hand grip strength on osteoporosis and falling risk: a Mendelian randomization study
Source: Front Endocrinol (Lausanne). 2024 Oct 2;15:1433805. doi: 10.3389/fendo.2024.1433805 (PMC11479888; doi:10.3389/fendo.2024.1433805)
Supplement: Supplementary file 2 [file Table2.docx]

**STROBE-MR checklist of recommended items to address in reports of Mendelian randomization studies**^1^ ^2^

| **Item No.** | **Section** | **Checklist item** | **Relevant text from manuscript** |
| --- | --- | --- | --- |
| 1 | **TITLE and ABSTRACT** | Indicate Mendelian randomization (MR) as the study’s design in the title and/or the abstract if that is a main purpose of the study | TITLE: The Genetic Causal Effect of Hand Grip Strength on Osteoporosis and Falling Risk: A Mendelian Randomization Study  ABSTRACT: The Mendelian randomization study was used to investigate the genetic causal effect of low hand grip strength on total body bone mineral density (BMD) at different ages, OP, and falling risk. |
|  | **INTRODUCTION** |  |  |
| 2 | **Background** | Explain the scientific background and rationale for the reported study. What is the exposure? Is a potential causal relationship between exposure and outcome plausible? Justify why MR is a helpful method to address the study question | Sarcopenia and OP are two disorders with similar risk factors and biological pathways 11. Bone and muscle interact closely with one another physically, chemically, and metabolically 12. OP and sarcopenia frequently coexist 13-15, and are strongly associated with frailty, falls, fractures, hospitalizations, and mortality 16-18. Current evidence also suggests that sarcopenia may be an independent predictor of low BMD and OP 13. Decreased hand grip strength is an important part of the diagnostic criteria for sarcopenia 19. Hand grip strength is the most preferable method of measuring muscle strength because it is a simple, noninvasive indicator of muscle strength and is ideal for clinical use 20. In recent years, some studies have shown that low hand grip strength could predict decreased bone mineral density (BMD) 13,21-23, the increased prevalence of OP 24,25, and falling risk 26,27, but the findings are inconsistent, and the limitations of observational studies make it unclear whether these associations are confounding or causal 28. Further research at the genetic level is required in order to fully understand the significance of these associations for disease prevention and screening.  Mendelian randomization (MR) is a method based on genome-wide association study (GWAS) data, where genetic variation is used as an instrumental variable (IV) to infer the specific effect of exposure on outcome 29. Genes are randomly assigned to the offspring without being subject to confounding factors because gamete formation follows Mendelian laws 30. To the best of our knowledge, no similar MR studies have been conducted to explore the causal relationship between hand grip strength and OP and fall risk. The MR study aimed to investigate the causal effect of low hand grip strength on the total body BMD at different ages, the prevalence of OP, and falling risk. |
| 3 | **Objectives** | State specific objectives clearly, including pre-specified causal hypotheses (if any). State that MR is a method that, under specific assumptions, intends to estimate causal effects | Mendelian randomization (MR) is a method based on genome-wide association study (GWAS) data, where genetic variation is used as an instrumental variable (IV) to infer the specific effect of exposure on outcome 29. Genes are randomly assigned to the offspring without being subject to confounding factors because gamete formation follows Mendelian laws 30. To the best of our knowledge, no similar MR studies have been conducted to explore the causal relationship between hand grip strength and OP and fall risk. The MR study aimed to investigate the causal effect of low hand grip strength on the total body BMD at different ages, the prevalence of OP, and falling risk. |
|  | **METHODS** |  |  |
| 4 | **Study design and data sources** | Present key elements of the study design early in the article. Consider including a table listing sources of data for all phases of the study. For each data source contributing to the analysis, describe the following: |  |
|  | a) | Setting: Describe the study design and the underlying population, if possible. Describe the setting, locations, and relevant dates, including periods of recruitment, exposure, follow-up, and data collection, when available. | The summary statistics of SNPs related to low hand grip strength, total body BMD at different ages, OP, and falling risk were extracted from the GWAS database (https://gwas.mrcieu.ac.uk), which is publicly available, and the detailed information is shown in Table S1. |
|  | b) | Participants: Give the eligibility criteria, and the sources and methods of selection of participants. Report the sample size, and whether any power or sample size calculations were carried out prior to the main analysis | Single nucleotide polymorphisms (SNPs) were used as IVs in the MR investigation to demonstrate a causal relationship between low hand grip strength and total body BMD, OP, falling risk. The summary statistics of SNPs related to low hand grip strength, total body BMD at different ages, OP, and falling risk were extracted from the GWAS database (https://gwas.mrcieu.ac.uk), which is publicly available, and the detailed information is shown in Table S1. |
|  | c) | Describe measurement, quality control and selection of genetic variants | In the MR analysis, IVs must meet the three key assumptions 30,31. First, SNPs are strongly associated with low hand grip strength. Second, SNPs shouldn't be associated with any confounders. Third, SNPs affect the outcome only via low hand grip strength (Graphical Abstract).  With P < 5×10-8 serving as the screening condition, SNPs for low hand grip strength were selected as IVs based on published data. At the same time, we excluded SNPs that were in linkage disequilibrium status (R2 < 0.001, aggregation window = 10,000kb) to ensure independence. Finally, we calculated the R2 and F statistic to evaluate the bias of the weak IVs using the following formula: R2 = 2 × EAF × (1-EAF) × β2, F = R2 (N-K-1) / [ (1-R2)], where N is the sample size, K is the number of IVs, and SNPs with F greater than 10 were further analyzed 32. Following a comprehensive screening process, the residual SNPs were employed in further investigations. |
|  | d) | For each exposure, outcome, and other relevant variables, describe methods of assessment and diagnostic criteria for diseases | The summary statistics of SNPs related to low hand grip strength, total body BMD at different ages, OP, and falling risk were extracted from the GWAS database (https://gwas.mrcieu.ac.uk), which is publicly available, and the detailed information is shown in Table S1. |
|  | e) | Provide details of ethics committee approval and participant informed consent, if relevant | Since this study was based on published data, no ethical approval or informed consent was required. |
| 5 | **Assumptions** | Explicitly state the three core IV assumptions for the main analysis (relevance, independence and exclusion restriction) as well assumptions for any additional or sensitivity analysis | In the MR analysis, IVs must meet the three key assumptions 30,31. First, SNPs are strongly associated with low hand grip strength. Second, SNPs shouldn't be associated with any confounders. Third, SNPs affect the outcome only via low hand grip strength. |
| 6 | **Statistical methods: main analysis** | Describe statistical methods and statistics used |  |
|  | a) | Describe how quantitative variables were handled in the analyses (i.e., scale, units, model) | The MR results were represented by odds ratios (ORs) and 95% confidence intervals (CIs). |
|  | b) | Describe how genetic variants were handled in the analyses and, if applicable, how their weights were selected | The random-effects inverse variance weighted (IVW) was the primary statistical method, which was used to analyze the primary causal inference of the effect of low hand grip strength on BMD, OP, and falling risk. To improve the confidence of the results, we used two additional MR methods, the weighted median and MR-Egger methods for causal association assessments. |
|  | c) | Describe the MR estimator (e.g. two-stage least squares, Wald ratio) and related statistics. Detail the included covariates and, in case of two-sample MR, whether the same covariate set was used for adjustment in the two samples | The MR results were represented by odds ratios (ORs) and 95% confidence intervals (CIs). P < 0.05 was statistically significant. |
|  | d) | Explain how missing data were addressed |  |
|  | e) | If applicable, indicate how multiple testing was addressed | The random-effects inverse variance weighted (IVW) was the primary statistical method, which was used to analyze the primary causal inference of the effect of low hand grip strength on BMD, OP, and falling risk. To improve the confidence of the results, we used two additional MR methods, the weighted median and MR-Egger methods for causal association assessments. In addition, we performed a series of sensitivity analyses to assess the reliability of the MR results. The Cochran’s Q test was used to detect heterogeneity of IVs. The MR-Egger intercept test and MR-PRESSO global test were used to examine the horizontal pleiotropy, and a leave-one-out sensitivity analysis was performed to assess the stability of the MR results. |
| 7 | **Assessment of assumptions** | Describe any methods or prior knowledge used to assess the assumptions or justify their validity | Mendelian randomization (MR) is a method based on genome-wide association study (GWAS) data, where genetic variation is used as an instrumental variable (IV) to infer the specific effect of exposure on outcome 29. Genes are randomly assigned to the offspring without being subject to confounding factors because gamete formation follows Mendelian laws 30. |
| 8 | **Sensitivity analyses and additional analyses** | Describe any sensitivity analyses or additional analyses performed (e.g. comparison of effect estimates from different approaches, independent replication, bias analytic techniques, validation of instruments, simulations) | The random-effects inverse variance weighted (IVW) was the primary statistical method, which was used to analyze the primary causal inference of the effect of low hand grip strength on BMD, OP, and falling risk. To improve the confidence of the results, we used two additional MR methods, the weighted median and MR-Egger methods for causal association assessments. In addition, we performed a series of sensitivity analyses to assess the reliability of the MR results. The Cochran’s Q test was used to detect heterogeneity of IVs. The MR-Egger intercept test and MR-PRESSO global test were used to examine the horizontal pleiotropy, and a leave-one-out sensitivity analysis was performed to assess the stability of the MR results. |
| 9 | **Software and pre-registration** |  |  |
|  | a) | Name statistical software and package(s), including version and settings used | The “TwoSampleMR” and “MRPRESSO” packages of the R software (version 4.3.1) were performed to implement all statistical analyses. |
|  | b) | State whether the study protocol and details were pre-registered (as well as when and where) | Since this study was based on published data, no ethical approval or informed consent was required. |
|  | **RESULTS** |  |  |
| 10 | **Descriptive data** |  |  |
|  | a) | Report the numbers of individuals at each stage of included studies and reasons for exclusion. Consider use of a flow diagram | As shown in Table S2, in our MR study, seventeen SNPs were chosen as IVs for low hand grip strength from published data. |
|  | b) | Report summary statistics for phenotypic exposure(s), outcome(s), and other relevant variables (e.g. means, SDs, proportions) | 3.2 The influence of genetically predicted low hand grip strength on total body BMD at different ages |
|  | c) | If the data sources include meta-analyses of previous studies, provide the assessments of heterogeneity across these studies |  |
|  | d) | For two-sample MR:  i.  Provide justification of the similarity of the genetic variant-exposure associations between the exposure and outcome samples  ii.  Provide information on the number of individuals who overlap between the exposure and outcome studies |  |
| 11 | **Main results** |  |  |
|  | a) | Report the associations between genetic variant and exposure, and between genetic variant and outcome, preferably on an interpretable scale | As shown in Table S2, in our MR study, seventeen SNPs were chosen as IVs for low hand grip strength from published data, and the F of all SNPs was greater than 10, no bias was found for weak IVs. |
|  | b) | Report MR estimates of the relationship between exposure and outcome, and the measures of uncertainty from the MR analysis, on an interpretable scale, such as odds ratio or relative risk per SD difference | 3.2 The influence of genetically predicted low hand grip strength on total body BMD at different ages  According to IVW analysis, the MR results indicated low hand grip strength could not directly affect the different ages of total body BMD (BMD age 0-15: OR = 1.03, 95% CI: 0.88-1.22, p = 0.698; BMD age 15-30: OR = 0.96, 95% CI: 0.70-1.33, p = 0.808; BMD age 30-45: OR = 1.11, 95% CI: 0.92-1.33, p = 0.284; BMD age 45-60: OR = 0.89, 95% CI: 0.76-1.03, p = 0.125; BMD age over 60: OR = 1.01, 95% CI: 0.86-1.18, p = 0.916), and the results were also confirmed by the MR-Egger regression and weighted median methods (all P > 0.05), which are presented in Table 1, Figure 1 and Figure 2.  3.3 The influence of genetically predicted low hand grip strength on OP  As presented in Table 1, Figure 3, the MR results of IVW analysis indicated low hand grip strength could directly affect the OP (OR: 1.006, 95% CI: 1.003-1.010, P= 0.0001), and the results were also confirmed by the weighted median methods (OR = 1.007, 95% CI: 1.003-1.012, P= 0.421).  3.4 The influence of genetically predicted low hand grip strength on falling risk  According to IVW analysis, the MR results indicated low hand grip strength could directly affect the falling risk (OR: 1.069, 95% CI: 1.013-1.129; P=0.0160), which are presented in Table 1, Figure 4. |
|  | c) | If relevant, consider translating estimates of relative risk into absolute risk for a meaningful time period |  |
|  | d) | Consider plots to visualize results (e.g. forest plot, scatterplot of associations between genetic variants and outcome versus between genetic variants and exposure) | Figure 1 Scatter plot of the causal effect of low hand grip strength on different ages of total body bone mineral density  Figure 2 Leave-one-out plot of the causal effect of low hand grip strength on different ages of total body bone mineral density  Figure 3 The causal effect of low hand grip strength on osteoporosis risk.  Figure 4 The causal effect of low hand grip strength on falling risk. |
| 12 | **Assessment of assumptions** |  |  |
|  | a) | Report the assessment of the validity of the assumptions | As shown in Table S2, in our MR study, seventeen SNPs were chosen as IVs for low hand grip strength from published data, and the F of all SNPs was greater than 10, no bias was found for weak IVs. |
|  | b) | Report any additional statistics (e.g., assessments of heterogeneity across genetic variants, such as *I^2^*, Q statistic or E-value) | As is shown in Table 2, all p values for Cochran's Q test analysis were more than 0.05, indicating no heterogeneity in the study. Also, the P values of the MR‐Egger intercept test and MR-PRESSO global test were all greater than 0.05, indicating no horizontal pleiotropy (Table 2). Additionally, each SNP was gradually removed by using the leave-one-out method, and the results were all the same as the original results, which showed the results of the study to be of heightened reliability (Figure 2, Figure 3, and Figure 4) |
| 13 | **Sensitivity analyses and additional analyses** |  |  |
|  | a) | Report any sensitivity analyses to assess the robustness of the main results to violations of the assumptions | As is shown in Table 2, all p values for Cochran's Q test analysis were more than 0.05, indicating no heterogeneity in the study. |
|  | b) | Report results from other sensitivity analyses or additional analyses | Also, the P values of the MR‐Egger intercept test and MR-PRESSO global test were all greater than 0.05, indicating no horizontal pleiotropy (Table 2). |
|  | c) | Report any assessment of direction of causal relationship (e.g., bidirectional MR) |  |
|  | d) | When relevant, report and compare with estimates from non-MR analyses |  |
|  | e) | Consider additional plots to visualize results (e.g., leave-one-out analyses) | Additionally, each SNP was gradually removed by using the leave-one-out method, and the results were all the same as the original results, which showed the results of the study to be of heightened reliability (Figure 2, Figure 3, and Figure 4). |
|  | **DISCUSSION** |  |  |
| 14 | **Key results** | Summarize key results with reference to study objectives | Exploring the causal relationship between hand grip strength and OP and falling risk is important for the prevention and screening of OP and falling. Previous studies are contradictory and have limitations in study design. Our study was conducted by a MR analysis method utilizing publicly available large-scale GWAS summary data, which ultimately found that genetic susceptibility to hand grip strength directly altered the risk of OP and falling. To our knowledge, this is the first MR study exploring the causal effect of hand grip strength on OP and falling risk. |
| 15 | **Limitations** | Discuss limitations of the study, taking into account the validity of the IV assumptions, other sources of potential bias, and imprecision. Discuss both direction and magnitude of any potential bias and any efforts to address them | Nevertheless, our study has some limitations. Firstly, the OP cases were from self-reported OP patients in the UK Biobank. Since disease reporting accuracy varies, there is a risk that OP cases will be misdiagnosed or underdiagnosed. Secondly, we must recognize that there are some limitations to the key assumptions of MR, as it is difficult to ensure that the exposure-outcome relationship is free of any confounders or potential pleiotropic effects. In addition, the GWAS data of the total body BMD lacked gender stratification, preventing a more detailed analysis of the causal relationship between hand grip strength and BMD in different gender subgroups. Besides, we were unable to perform stratified MR analyses based on subtypes of OP, which would have helped to improve the accuracy of the study. Furthermore, although the MR method was used in this study to assess the causal relationship, it presupposes that there is a linear relationship between exposure and outcome, otherwise, this method is not applicable, so prospective cohort data are still needed to validate this in the future. |
| 16 | **Interpretation** |  |  |
|  | a) | Meaning: Give a cautious overall interpretation of results in the context of their limitations and in comparison with other studies | Our study was conducted by a MR analysis method utilizing publicly available large-scale GWAS summary data, which ultimately found that genetic susceptibility to hand grip strength directly altered the risk of OP and falling. To our knowledge, this is the first MR study exploring the causal effect of hand grip strength on OP and falling risk. |
|  | b) | Mechanism: Discuss underlying biological mechanisms that could drive a potential causal relationship between the investigated exposure and the outcome, and whether the gene-environment equivalence assumption is reasonable. Use causal language carefully, clarifying that IV estimates may provide causal effects only under certain assumptions | Bone and skeletal muscle are integral organs and the coupling between them is considered to be primarily mechanical 39. In addition to the direct effects of weight-bearing, physical activity is the main physiological stimulus that promotes skeletal anabolism and/or catabolism through actin production and secretion 39. However, skeletal muscle can also influence skeletal homeostasis in a non-mechanical way, i.e., through its endocrine activity, actin secreted by skeletal muscle has not only an autocrine function in regulating muscle metabolism but also a paracrine or endocrine regulatory function in distant organs and tissues such as bone and adipose tissue 39,40. Age-related skeletal muscle decline may lead to bone loss through biomechanical stimulation and decreased growth factors, ultimately leading to the development of OP 41. As a result, patients with sarcopenia have an increased likelihood of developing OP, and some experts have suggested that the two disorders should be combined into a single disease called “osteosarcopenia” 12.  Although the MR method was used in this study to assess the causal relationship, it presupposes that there is a linear relationship between exposure and outcome, otherwise, this method is not applicable, so prospective cohort data are still needed to validate this in the future.  Secondly, we must recognize that there are some limitations to the key assumptions of MR, as it is difficult to ensure that the exposure-outcome relationship is free of any confounders or potential pleiotropic effects. |
|  | c) | Clinical relevance: Discuss whether the results have clinical or public policy relevance, and to what extent they inform effect sizes of possible interventions | Exploring the causal relationship between hand grip strength and OP and falling risk is important for the prevention and screening of OP and falling. Previous studies are contradictory and have limitations in study design. Our study was conducted by a MR analysis method utilizing publicly available large-scale GWAS summary data, which ultimately found that genetic susceptibility to hand grip strength directly altered the risk of OP and falling. To our knowledge, this is the first MR study exploring the causal effect of hand grip strength on OP and falling risk.  In summary, our study provides genetic evidence to support a causal association between low hand grip strength and OP, fall risk. Hand grip strength measurement is a simple, cost-effective, and easy-to-administer assessment method for identifying people at high risk for OP and falls, which should be taken into account in the development of future prevention and screening strategies for the disease. |
| 17 | **Generalizability** | Discuss the generalizability of the study results (a) to other populations, (b) across other exposure periods/timings, and (c) across other levels of exposure | Nevertheless, our study has some limitations. Firstly, the OP cases were from self-reported OP patients in the UK Biobank. Since disease reporting accuracy varies, there is a risk that OP cases will be misdiagnosed or underdiagnosed. Secondly, we must recognize that there are some limitations to the key assumptions of MR, as it is difficult to ensure that the exposure-outcome relationship is free of any confounders or potential pleiotropic effects. In addition, the GWAS data of the total body BMD lacked gender stratification, preventing a more detailed analysis of the causal relationship between hand grip strength and BMD in different gender subgroups. Besides, we were unable to perform stratified MR analyses based on subtypes of OP, which would have helped to improve the accuracy of the study. Furthermore, although the MR method was used in this study to assess the causal relationship, it presupposes that there is a linear relationship between exposure and outcome, otherwise, this method is not applicable, so prospective cohort data are still needed to validate this in the future. |
|  | **OTHER INFORMATION** |  |  |
| 18 | **Funding** | Describe sources of funding and the role of funders in the present study and, if applicable, sources of funding for the databases and original study or studies on which the present study is based | This work was supported by National High Level Hospital Clinical Research Funding (BJ-2021-200, BJ-2022-193 and BJ-2022-120), National Natural Science Foundation of China (82170848), Capital’s Funds for Health Improvement and Research (2022-1-4051) and Beijing Municipal Science & Technology Commission No Z221100007422007. |
| 19 | **Data and data sharing** | Provide the data used to perform all analyses or report where and how the data can be accessed, and reference these sources in the article. Provide the statistical code needed to reproduce the results in the article, or report whether the code is publicly accessible and if so, where | The datasets for this study can be found in the GWAS public database (<https://gwas.mrcieu.ac.uk>).  The summary statistics of SNPs related to low hand grip strength, total body BMD at different ages, OP, and falling risk were extracted from the GWAS database (https://gwas.mrcieu.ac.uk), which is publicly available, and the detailed information is shown in Table S1. |
| 20 | **Conflicts of Interest** | All authors should declare all potential conflicts of interest | The authors declare that the research was conducted in the absence of any commercial or financial relationships that could be construed as a potential conflict of interest. |

This checklist is copyrighted by the Equator Network under the Creative Commons Attribution 3.0 Unported (CC BY 3.0) license.

1. Skrivankova VW, Richmond RC, Woolf BAR, Yarmolinsky J, Davies NM, Swanson SA, et al. Strengthening the Reporting of Observational Studies in Epidemiology using Mendelian Randomization (STROBE-MR) Statement. JAMA. 2021;under review.

2. Skrivankova VW, Richmond RC, Woolf BAR, Davies NM, Swanson SA, VanderWeele TJ, et al. Strengthening the Reporting of Observational Studies in Epidemiology using Mendelian Randomisation (STROBE-MR): Explanation and Elaboration. BMJ. 2021;375:n2233.
